# Complete Genome Sequence of Bacillus safensis Strain AHB2, Isolated from African Raw Honey from Kajo Keji, South Sudan

**DOI:** 10.1128/mra.00515-22

**Published:** 2022-10-20

**Authors:** Carson J. Shin, Tamara J. O’Connor

**Affiliations:** a Department of Biological Chemistry, The Johns Hopkins University School of Medicine, Baltimore, Maryland, USA; University of Rochester School of Medicine and Dentistry

## Abstract

We report the complete genome sequence of Bacillus safensis strain AHB2, isolated from African raw honey originating in Kajo Keji, South Sudan, and purchased from a third-party vendor. The genome consists of 3,785,324 bp encompassing 3,774 predicted protein-coding sequences and 183 RNA genes.

## ANNOUNCEMENT

Natural honey is highly regarded for its medicinal utility due to its potent antimicrobial activity against many bacterial pathogens (1–12). Thus, honey is generally regarded as relatively sterile, with only a few spore-forming microorganisms able to survive within honey (13–20). In this study, we present the complete genome sequence of a bacterium isolated from African raw honey from Kajo Keji, South Sudan, identified as Bacillus safensis and subsequently named strain AHB2, for “African honey bacterial isolate 2.”

The bacterium was isolated and cultivated by diluting honey 50% (vol/vol) in sterile water, plating on solid ACES [*N*-(2-acetamido)-2-aminoethanesulfonic acid]-buffered charcoal yeast extract (CYE) medium (21) containing 12 g/L agar and supplemented with 0.4 g/L iron(III) nitrate, 0.135 g/L cysteine, and 0.1 mg/mL thymidine, and growing at 37°C for 20 h. For DNA isolation, bacteria were cultured from a single colony in liquid CYE medium at 37°C with aeration for 20 h.

Bacterial DNA was isolated using a DNeasy blood and tissue kit (Qiagen) following the manufacturer’s instructions, with the following modifications for bacterial lysis. Bacteria were resuspended in 100 μL of lysis buffer (20 mM Tris-HCl [pH 8], 2 mM EDTA, 1.2% Triton X-100) containing 20 mg/mL lysozyme and incubated for 3 h at 37°C. Samples were subsequently treated with proteinase K in Qiagen AL buffer for 2 h at 56°C. DNA quantity and quality were determined using a Tecan spectrophotometer fitted with a NanoQuant microplate.

Genome sequencing and assembly were performed at the Microbial Genome Sequencing Center (MiGS) (Pittsburgh, PA, USA). Samples for Illumina sequencing were prepared using the Illumina DNA preparation kit, including fragmentation and size selection (average 320 bp). Paired-end sequencing (150 bp) was performed on an Illumina NextSeq2000 instrument. Read quality (Q30 or higher) and adapter trimming were performed with bcl2fastq v2.20.0.445 (22). Samples for Oxford Nanopore Technologies (ONT) sequencing were prepared using the genomic-DNA-by-ligation kit (Oxford Nanopore Technologies) following the manufacturer’s instructions. Sequencing was performed on a Nanopore MinION R9.4.1 flow cell. Read quality (Q30 or higher) and adapter trimming were performed using Guppy v5.0.16 (Nanopore Technologies) in high-accuracy base calling mode and Porechop v0.2.3_seqan2.1.1 (23). Illumina and ONT (*N*_50_ = 1,044) sequencing resulted in 2,503,986 and 233,309 reads, respectively, with 86× coverage. Genome assembly and circularity determination were performed with Unicycler v0.4.8 (24), and assembly statistics were assessed with QUAST v5.0.2 (25). For all software, default parameters were used unless otherwise specified. The highest percent sequence homology of 16S, 23S, *rpoB*, *gyrB*, and *recA* genes defined by BLAST (26) was used to determine bacterium identity.

Bacillus safensis strain AHB2 had a genome of 3,785,324 bp and a GC content of 41.7%. The genome was annotated by NCBI using the Prokaryotic Genome Annotation Pipeline (PGAP) (27), which identified 3,895 genes encompassing 3,786 coding DNA sequences (CDSs), 24 rRNAs, 80 tRNAs, 1 transfer-messenger RNA (tmRNA), and 4 noncoding RNAs.

### Data availability.

The complete genome sequence of Bacillus safensis strain AHB2 was deposited in GenBank under the accession number CP097373. The associated BioProject and BioSample accession numbers are PRJNA836920 and SAMN28178808, respectively.
